# Predicted Relative Metabolomic Turnover (PRMT): determining metabolic turnover from a coastal marine metagenomic dataset

**DOI:** 10.1186/2042-5783-1-4

**Published:** 2011-06-14

**Authors:** Peter E Larsen, Frank R Collart, Dawn Field, Folker Meyer, Kevin P Keegan, Christopher S Henry, John McGrath, John Quinn, Jack A Gilbert

**Affiliations:** 1 Argonne National Laboratory, 9700, S. Cass Ave, Argonne, Illinois, USA; 2 NERC Centre for Ecology and Hydrology, CEH Oxford, Mansfield Road, Oxford, OX1 3SR, UK; 3 School of Biological Science, Queens University, Medical Biology Centre, 97 Lisburn Road. Belfast, BT9 7BL, Northern Ireland, UK; 4 Computation Institute, University of Chicago, 5735 South Ellis Avenue, Chicago, IL 60637, USA; 5 Department of Ecology and Evolution, University of Chicago, 5640 South Ellis Avenue, Chicago, IL 60637, USA

## Abstract

**Background:**

The world's oceans are home to a diverse array of microbial life whose metabolic activity helps to drive the earth's biogeochemical cycles. Metagenomic analysis has revolutionized our access to these communities, providing a system-scale perspective of microbial community interactions. However, while metagenome sequencing can provide useful estimates of the relative change in abundance of specific genes and taxa between environments or over time, this does not investigate the relative changes in the production or consumption of different metabolites.

**Results:**

We propose a methodology, Predicted Relative Metabolic Turnover (PRMT) that defines and enables exploration of metabolite-space inferred from the metagenome. Our analysis of metagenomic data from a time-series study in the Western English Channel demonstrated considerable correlations between predicted relative metabolic turnover and seasonal changes in abundance of measured environmental parameters as well as with observed seasonal changes in bacterial population structure.

**Conclusions:**

The PRMT method was successfully applied to metagenomic data to explore the Western English Channel microbial metabalome to generate specific, biologically testable hypotheses. Generated hypotheses linked organic phosphate utilization to *Gammaproteobactaria*, *Plantcomycetes*, and *Betaproteobacteria*, chitin degradation to *Actinomycetes*, and potential small molecule biosynthesis pathways for *Lentisphaerae*, *Chlamydiae*, and *Crenarchaeota*. The PRMT method can be applied as a general tool for the analysis of additional metagenomic or transcriptomic datasets.

## Background

Marine biomes dominate the planet's surface and single-celled microorganisms are responsible for up to 98% of the ocean's primary productivity [1]; understanding the nutrient and carbon cycles of the world's oceans has key applications for understanding global ecology. The extremely diverse marine microbial communities mediate the largest active pool of near-surface carbon on the planet [2] and are a dominant force in the planet's biogeochemical cycles [3]. The L4 Station of the Western Channel Observatory (WCO), an oceanographic time-series and marine biodiversity reference site in the Western English Channel http://www.westernchannelobservatory.org.uk, provides a unique opportunity to study a coastal marine microbial ecosystem. Environmental parameter data from the WCO have been continuously monitored for over a century. More recently, microbial metagenomic data collected from this site have shown that the abundance and relative composition of genes and taxa change over time, demonstrating seasonal structure and predictable community responses to environmental parameters [4-7].

The seasonal structure in the community composition of both taxa and genes has potential repercussions for the seasonal succession in metabolic potential, which will drive the range and relative abundance of metabolites produced and consumed by a community. Metagenomic analyses explore the functional potential of an ecosystem by describing the changes in the abundance of genes annotated with unique enzyme functions. Here we propose a methodology that alters this paradigm, by describing the metagenome in terms of the relative change in the production or consumption of specific metabolites. Instead of exploring gene-space using an environmental metagenomic analysis, this study explores derived metabolite-space, inferred from the metagenome. The predicted environmental metabolome is the set of all detected unique enzyme functions encoded in a metagenome and all of the metabolites implied by those activities. This network of predicted metabolic reactions represents the theoretical metabolic potential of an environmental metabolome and provides a novel means through which metagenomic observations of seasonal and/or biogeographic trends in microbial communities can be utilized to explore community-wide metabolic dynamics.

Understanding the metabolomic interactions in any marine microbial community is a daunting task. Classical metabolomic techniques such as NMR or GC-MS [8-11], while powerful, provide measurements for just a fraction of the metabolites predicted to be present in a metabolome. As it is not possible to easily measure every metabolite in an environment, it is important to determine the most pertinent parameters, those that will allow investigators to generate testable biological hypotheses. Currently, methods are available to extract a wide variety of environmental features from metagenomic sequence data [12-14]. Metagenomics can be used to determine the taxonomic and functional diversity of a microbial community via automated pipelines [15] to curated protein databases such as RefSeq [16], KEGG [17], KEGGnoggs [18], SEED [19], PFAM [20] or TIGRfam [21], and linking environmental conditions with specific metabolic activities inferred from metagenomic data [22].

Here, we propose a novel computational method, Predicted Relative Metabolic Turnover (PRMT), which enables comparative analyses of environmental metabolomes predicted from metagenomic data. To support the methodology with specific biological observations, PRMT was applied to a metagenomic dataset from the time-series study of the bacterial environmental metabolome in the Western English Channel [4]. The PRMT approach also correctly predicted considerable correlations between environmental metabolite concentrations and PRMT metabolomic predictions. It also correctly predicting seasonal variations in the bacterial community primary productivity, and generated specific, testable biological hypotheses for organic phosphate utilization by *Gammaproteobactaria*, *Plantcomycetes*, and *Betaproteobacteria*; chitin degradation by *Actinomycetes*; and potential small molecule biosynthesis pathways for *Lentisphaerae*, *Chlamydiae*, and *Crenarchaeota*.

## Materials and methods

### Data used for validation of methodology

Metagenomic sequencing data was acquired from a recent study examining the seasonal structure of functional potential in the Western English Channel [4]. Specific data points for use in this study came from day/night pairs of samples taken on January 28, April 27, and August 27 2008. All data collected on the same day were averaged; previous analyses of these data have shown that the metagenomic functional gene profile is statistically identical between day and night in each of these samples [4]. All metagenomic data were annotated with MG-RAST [15] using parameters previously described [4]. Specifically, nucleic acid sequences were excluded if annotated as rRNA, and all subsequent reads were annotated against the SEED database using MG-RAST (e-value < 1 × 10-3; minimum length of alignment of 50 bp; minimum sequence nucleotide identity of 50%; [15]) to produce an abundance matrix of functional genes and protein-derived predicted taxonomies across the DNA samples. Short read data are available through the European Nucleotide Archive (ENA) short read archive under ERP000118 http://www.ebi.ac.uk/ena/data/view/ERP000118. All data are available on the CAMERA website under 'Western Channel Observatory Microbial Metagenomic Study' http://camera.calit2.net. MG-RAST annotations of metagenomic data were collected under IDs 4445064.3, 444077.3, 4445065.3, 4445066.3, 4445068.3, 4444083.3, 4445069.3, and 4445070 http://metagenomics.anl.gov/. All submissions conform to the minimum information standards (MIxS) of the Genomic Standards Consortium [23].

### PRMT analysis approach

PRMT scores predict the change in turnover of metabolites (defined as the potential for consumption or production) in an environmental metabolome, given the relative abundance of genes for unique enzyme functions detected in different metagenomes. In this manuscript, we use the term "unique enzyme function" to describe a specific annotation applied to an enzyme, i.e. "Phosphotransferases with an alcohol group as acceptor". We use "enzyme reactions" to refer to metabolite transformations catalyzed by an enzyme function, i.e "ATP + D-Glycerate ↔ ADP + 3-Phospho-D-glycerate". A unique enzyme function may catalyze more than one enzyme reaction and an enzyme reaction may be catalyzed by more than one unique enzyme function. A metabolite is a molecular compound that is a reactant or product in an enzyme reaction. In PRMT, a metabolite is never the protein product of a gene in the metagenome.

**Figure 1:**
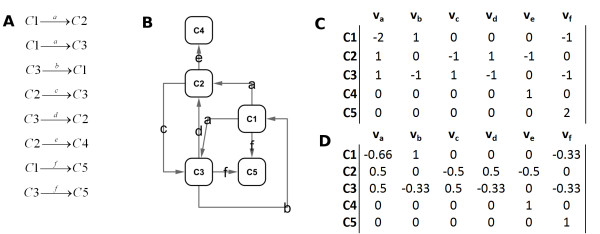
**Example of generating an EMM from metagenomic data**. This figure is an example of generating a simple EMM with hypothetical data. Letters a-f represent unique enzyme functions identified in the annotation of a hypothetical set of metagenomes. In (A), the set of all enzyme reactions for enzyme functions a-f between compounds C1-C5 from a database of possible reactions is listed. In (B), a metabolome is constructed from the reactions identified in A. (C) Shows the connectivity matrix of the network in B. (D) Is the complete EMM for metagenomic annotated enzyme functions a-f, normalizing values in C such that the sum of all inputs to a compound is 1 and the sum of all outputs from a compound is -1.

This method makes a number of assumptions. First, as with many metagenomic analyses, it assumes that relative abundance of genes for a unique enzyme function in metagenomic sequence is proportionate to relative abundance of expressed functional proteins. Second, PRMT assumes the rate of a reaction is proportionate to the amount of enzyme, and not to the concentrations of reactant or product. Finally, PRMT assumes that the marine metabolome can be modeled as a well-mixed reaction, disregarding compartmentalization of metabolites and activities within individual bacteria. All unique enzyme functions annotated to a set of metagenomes are compared to reference databases of enzyme reactions to infer the set of metabolites present. Below we describe the three main steps to calculating PRMT scores (Figure 1):

(1) *Generate Environmental Metabolome Matrix (EMM*). A network, constructed from annotated unique enzyme functions, enzyme reactions and inferred metabolites, is used to generate the predicted environmental metabolome; this network is expressed as a connectivity matrix, the Environmental Metabolomic Matrix (EMM; Figure 1). The resulting EMM matrix has dimensions *n *x *m*, where *n *is the number of predicted metabolites in the environmental metabolome and *m *is the number of unique enzyme functions detected in the set of analyzed metagenomes. The set of Kyoto Encyclopedia of Genes and Genomes (KEGG) [17] metabolic reactions was used to represent the set of possible enzyme reactions in an environmental metabolome and Enzyme Commission (EC) number annotations for enzyme activity from MG-RAST annotations of metagenomic sequences were used to assign unique enzyme functions to the predicted proteins encoded in a metagenome. KEGG reactions used were selected exclusively from KEGG 'Metabolism' pathways. KEGG pathway maps that are not directly related to specific metabolic activities in microorganisms (e.g. Human Diseases, Drug Development, etc.) were not used in generation of metabolomes. A complete list of the 156 KEGG pathways used in construction of EMM is provided as supplementary data [Additional file 1, Table S1]. As is common in network analysis of biochemical systems [24], the KEGG metabolites water, di- and tri-phosphonucleotides, and all ubiquitous cofactors were excluded from the list of possible reactants as non-specific to particular reactions and metabolic processes. If a reaction is identified as reversible in the KEGG database, then both forward and reverse reactions are included in the EMM. If a particular metabolite transformation is attributed to more than one enzyme activity, then each transformation reaction is incorporated into the EMM. The stoichiometry of each reaction was not considered as the quantity of metabolites was not considered, just the relative turnover of each.

(2) *Generate normalized Enzyme Activity Counts (EACs)*. For each unique enzyme function in each metagenome in the EMM, an Enzyme Activity Count (EAC) is determined by the following equation:

*EAC_i _*is the Enzyme Activity Count for enzymatic function *i*, and *N_seq,i _*is the number of sequence reads in the metagenome annotated with a unique enzyme function *i*. Collected EACs for a set of metagenomes are normalized (nEAC) by quantiles [25]. Quantile normalization is a technique for making distributions from multiple datasets identical in statistical properties. To quantile-normalize the sets of EACs, the EACs are sorted largest to smallest. A reference distribution is made from the sorted lists such that the highest value in all cases becomes the mean of the highest values, the second highest value becomes the mean of the second highest values, and so on. For each set of EACs, nEAC is generated by assigning the distributions of the reference distribution to the observed EAC distribution. The set of nEACs for a metagenome is expressed as a vector of length *n*, where *n *is the total number of unique enzyme functions found in the set of metagenomes.

(3) *Calculate PRMT-scores*. A PRMT-score is calculated for each metabolite in the EMM in a metagenome using the following equation:

 is a vector of PRMT-scores of length *m*, where *m *is the number of metabolic compounds in the EMM.  and  are vectors of normalized enzyme gene counts (nEAC) of length *n*, where *n *is the number of unique enzyme functions annotated to metagenomic sequences, and *x *and *y *refer to different metagenome datasets. *M *is the EMM, a matrix of dimensions *m *x *n*. Changes in nEAC for a reversible interaction do not change calculated PRMT-scores for metabolites. For analysis of the L4 WCO environmental metabolome, the reference nEAC was the average of EAC's across all samples from January, April, and August.

### Interpretation of PRMT scores

PRMT-scores are unit-less values that represent the change of the turnover of a metabolite in a predicted metabolome relative to a reference metabolome. A PRMT-score is calculated for every metabolite in the EMM for a metagenome. The value and sign (positive or negative) of a PRMT-score provides information about a metabolite's relative turnover. Although a thorough interpretation of a PRMT-score requires that it be considered in the greater context of the complete network, it can be broadly interpreted as follows: A positive PRMT score predicts increased metabolic turnover and relatively greater consumption of a metabolite. A negative PRMT-score predicts decreased turnover and relatively greater accumulation of a metabolite. It is important to note that PRMT-scores do not predict net production or consumption of a metabolite.

### Correlations between PRMT-scores and environmental parameters

Numerous methods were considered for exploring the correlative relationships between calculated PRMT scores and measured environmental variables. The non-parametric Spearman's rank correlation was rejected because the resulting coefficients were considered too granular to produce appropriate interpretation of dataset with only 3 time points. Specifically, Spearman's rank correlation returned only 6 possible values of rho for all comparisons in this dataset. Hence, while Spearman's rank may well be suited to correlation coefficient calculation from a dataset with more time points, it was inappropriate for the current study. However, as the PRMT scores generated an approximately normal distribution, it was considered absolutely valid to utilize a parametric test for correlation, namely Pearson's Correlation Coefficient (PCC). The specific biological parameters considered were environmental metabolites ([4], Table 1, chlorophyll A, Total Organic Nitrogen (TON), Total Organic Carbon (TOC), NO2+NO3, Ammonia, and Soluble Reactive Phosphate (SRP)), bacterial phyla percent abundances [[7], Table S2] (only the 23 bacterial phyla and class present at a percent abundance of at least 1% of the total community abundance were used in this analysis.), and number of sequences in metagenomic data annotated to SEED hierarchy I subsystem relative abundances ([[4], Figure Seven A]).

**Table 1:** Correlations of calculated PRMT scores with relative abundance of selected environmental parameter measurements.

Parameter	PRMT metabolite	PCC
Chlorophyll A	Chlorophyll A	**-0.98**
Total Organic Nitrogen	alpha-Amino acid	**-0.99**
Total Organic Carbon	Starch	-0.98
NO_2_+NO_3_	Nitrite	**-0.98**
NH_3_	NH3	-0.81
Soluble Reactive Phosphorus	Orthophosphate	-0.93

Parameters are relative concentrations of biological measurements from Western English Channel L4 station and PRMT-metabolites are those predicted metabolites that are representative of measured parameters. PCC-scores between measured parameters and PRMT-scores are highlighted in **bold **if a strong correlation (i.e. in the top or bottom 5^th ^percentile of randomized resamples) was observed.

To enable correlation with PRMT scores, the environmental parameters, bacterial phyla percent abundances, and SEED subsystem relative abundances were converted to measures of log relative abundance. Log relative abundance of a parameter was calculated using the following equation:

*x *is a measured experimental parameter and  is the average for the parameter across all samples.

PCCs were calculated between four different combinations, namely; all measured environmental metabolites and metagenomic reads annotated to SEED subsystems; environmental metabolites and bacterial phyla; environmental metabolites and PRMT-scores; and PRMT-scores and bacterial phyla. It was considered that statistical significance could not be reliably assigned due to the small number of samples used in the analysis (3 seasonal time points). This was of particular concern given the method's reliance on a multiple-test based procedure. In order to address these concerns, and to provide an informal confidence estimate with which to judge each individual PCC, 10,000 randomized re-samplings of the initial data were used to generate a distribution of PCC-scores. Observed PCC-scores that were in the top or bottom 5th percentile of randomized re-sampled were considered to be a strong correlation.

A graphical representation of observed correlations was performed using 'Cytoscape' [51] to generate a network in which experimental measurements are represented as nodes and strong correlations between relative abundance of measurements were represented as edges.

## Results and Discussion

### Correlation networks between environmental parameters, bacterial phyla, and SEED subsystems

**Figure 2:**
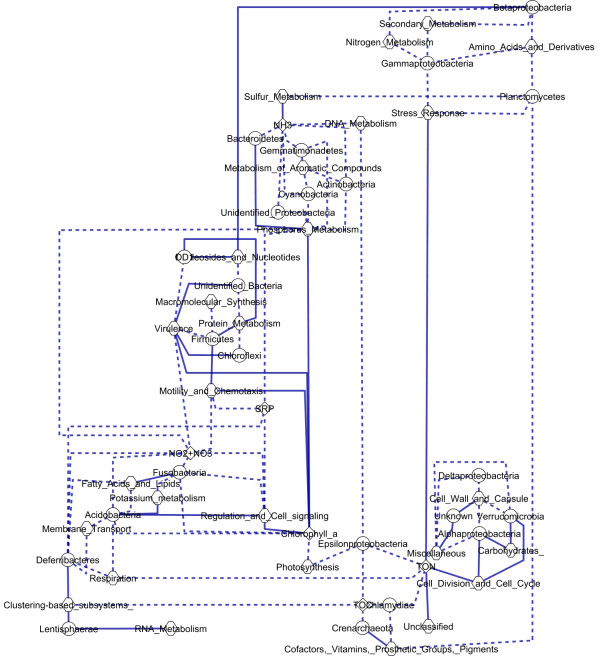
**Strong correlations between environmental metabolites, metabolic subsystems, and bacterial population structure**. This network is a graphical representation of strong (i.e. in the top or bottom 5^th ^percentile of randomized resamples) correlations between relative abundance of measured environmental metabolites (diamonds), relative abundance of metagenomic reads annotated to metagenomic SEED subsystems (hexagons), and relative abundance of bacterial taxa (circles) across seasonal variation for the Western English Channel L4 station. Strong positive correlations are represented by solid lines and strong negative correlations by dashed lines.

To demonstrate why PRMT demonstrates a significant advance on existing metagenomic analytical tools, e.g. exploring changes in the relative abundance of predicted gene functions, it was necessary to perform correlative network analysis of the relationships between the relative abundance of taxa, annotated genes and measured environmental parameters (Figure 2). Although there may be other environmental causal factors at play, this figure identifies correlations between changes in metabolic functions and environmental conditions or bacterial relative abundance. The most abundant taxa in this ecosystem, *Alphaproteobacteria*, had a strong positive correlation (in the 5^th ^percentile of randomized resamples) with the relative abundance of metagenomic reads annotated to SEED subsystems 'carbohydrate metabolism' and 'cell division'. Additionally, 'cell division' had a strong positive correlation with the relative abundance of TON. This suggests a relationship between TON availability and the growth of *Alphaproteobacterial *populations. Additionally, there is a strong positive correlation between the *Roseobacteriales *order of class *Alphaproteobacteria *and the availability of TON; both peak in the summer. The second most abundant phylum, *Bacteroidetes*, had a strong positive correlation with the SEED subsystem 'Phosphorus metabolism', which, as with TON and *Alphaproteobacteria*, could implicate Phosphorus as a limiting nutrient for *Bacteroidetes*. Interestingly, less abundant taxa were more frequently characterized by strong negative correlations with measurements of nutrients in their environment. The relative abundances of taxa *Chlamydiae*, *Crenarchaeota*, and *Epsilonpreoteobacteria *demonstrated a strong negative correlation with TON; *Deferribacteres *and *Fusobacteria *had a strong negative correlation with NO_2_+NO_3_; the phylum *Actinobacteria *had a strong negative correlation with ammonia. Conversely, rare phyla more frequently had strong positive correlations with SEED subsystems, *e.g*. 'Cofactors', 'vitamins', 'prosthetic groups, and pigments', 'Clustering based subsystems', 'Virulence', and 'Motility and chemotaxis'; further exploration of these may yield additional avenues of discovery.

A strong negative correlation was observed between the relative abundance of the SEED subsystem 'Photosynthesis metabolism' and the concentration of chlorophyll A. This is similar to previous observations [4] that indicate the relative abundance of genes with homology to components of the cyanobacterial photosynthesis pathway peak in winter, while chlorophyll A concentrations, which relate to Eukaryotic phytoplankton abundance, peak in summer. There is also a strong negative correlation between the relative change in concentration of SRP and the SEED subsystem for 'Phosphate metabolism'. This suggests that when there are more genes for phosphate metabolism there is a greater predicted likelihood for SRP consumption. As a caveat for all of these relationships, correlation does not prove causality; they do however, identify relationships that invite further investigation.

The analysis of the relationships between phyla, environmental parameters, and functional genes indicates that changes in taxonomic diversity affect environmental metabolomic functional potential. To complement this gene-centric perspective, we present a metabolite-centric tool, PRMT, which infers metabolic activity from unique enzyme functions.

### The Environmental Metabolomic Matrix from the Western English Channel

**Figure 3:**
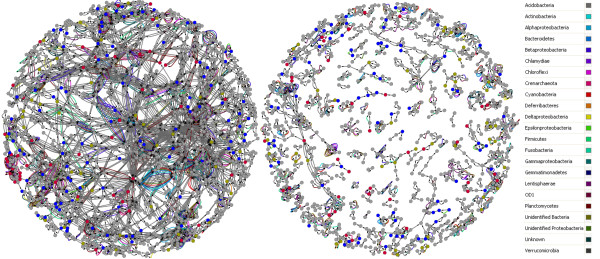
**L4 Environmental Metabolome**. In the figure, edges represent enzyme functions identified in annotated metagenomes. Nodes are predicted metabolites, inferred by the reactions catalyzed by detected enzyme functions. Nodes are highlighted if calculated PRMT scores for seasonal metagenomes correlate strongly (i.e. in the top or bottom 5^th ^percentile of randomized resamples) with relative abundance of measured environmental parameters (Red for Total Organic Carbon, blue for Total Organic Nitrogen, and gold for Soluble Reactive Phosphorus). Edges are highlighted in one of 23 colors if they connect nodes that correlate with relative abundance of a bacterial phylum.

The predicted L4 Western English Channel metabolome consists of 2281 predicted metabolites and 4152 enzyme reactions for 990 unique enzyme activities (Figure 3). In the EMM, 70% of enzyme reactions are reversible. The largest connected subnetwork in the network has 1257 metabolites and 3197 enzyme reactions. The second largest subnetwork contains 30 predicted metabolites and 51 enzymatic reactions. There are 194 subnetworks that consist of only two metabolites each. Approximately 63% of the enzyme activities in the KEGG global metabolism map (KEGG map01100) are present in the predicted environmental metabolome. Individual unique enzyme activities in map01100 were represented an average of 128 counts per metagenomic dataset. An average of 81% of total unique enzyme counts in each metagenome could be mapped to KEGG pathway map01100. The representation of core metabolic pathways in annotated metagenomes is especially evident within specific sub-groups; for example energy metabolism is well represented, with 95% of citrate cycle (map00020), 80% of glycolysis/gluconeogenesis (map00010), and 92% of photosynthesis metabolism (map 00195) enzyme activities detected. Cell membrane metabolism is detected as present in the predicted environmental metabolome, with 72% of fatty acid metabolism (map00071) and 65% of fatty acid biosynthesis (map00061). For the DNA metabolism, 65% of pyrimidine metabolism (map00240) and 67% of purine metabolism (map00230) are detected. For protein metabolism, 79% of glycine, serine, threonine metabolism (map00260), 89% of valine, leucine, and isoleucine biosynthesis (map00290), and 90% of phenylalanine, tyrosine, and tryptophan biosynthesis (map00400) enzyme activities are represented. For a bacteria-dominated system, 100% coverage of core metabolic KEGG pathways is not anticipated, as the database also contains plant and animal specific reactions.

Figure was generated using Cytoscape v2.6.1 [51]. The network and calculated PRMT-scores in this figure is available for download as Additional file 2, figure S1.

### PRMT scores calculated from the Western English Channel EMM

PRMT scores were calculated for each predicted metabolite in the EMM at each of three seasonal time points (January 28, April 27, and August 27, 2008). Correlations between PRMT-scores and relative abundance of environmental parameters were generated (Table 1). The complete set of PRMT-scores is available as supplemental data (Additional file 3, Table S2). A strong negative correlation between PRMT scores and relative changes in the concentration of an environmental parameter demonstrates that the metabolomic capacity for *synthesis *of a parameter increased when the parameter is at a relatively higher concentration. Conversely, a strong positive correlation occurs if metabolomic capacity for the *consumption *of a metabolite increases when the environmental parameter is at a relatively higher concentration. Only three of the six correlations were considered strong (i.e. in the top or bottom 5^th ^percentile of randomized resamples). However, using a calculation for the cumulative normal distribution (CND), given the distribution of correlations between relative abundances of environmental parameters and all calculated PRMT-scores, the probability that all six PCC-scores would be between -1 and -0.8 was < 1 × 10^-256 ^(lowest is -0.81; Table 1). This probability indicates that, even though some individual correlations are not strong (based on the given criteria), it is extremely unlikely that all six correlations could all be between -1 and -0.8 by chance, assuming a normal distribution of correlation coefficients.

Correlations between PRMT-scores and the relative abundances of bacterial phyla can be used to generate hypotheses regarding the potential of different taxonomic groups to produce specific metabolites in subnetworks in the EMM. For example, *Actinobacteria *play an important environmental role in the decomposition of cellulose [26]. The relative abundance of *Actinobacteria *had a strong negative correlation with PRMT-scores for the predicted metabolite cellulose. This strong negative correlation indicates that when the *Actinobacteria *are relatively more abundant, the predicted tendency for cellulose to be consumed in the environmental metabolome increases. In another example, the *Planctomycetes *was the only phyla whose change in relative abundance demonstrated a strong positive correlation with PRMT-scores for L-Glutamate. This was expected, as the cell wall scaffolds of most *Plancotomycetes *are made up of glycoproteins rich in glutamate instead of murein, as is common for other bacterial taxa [27]. Of greater interest than investigating the predicted metabolome for individual metabolites is to seek connected metabolic subnetworks in the EMM that have strong correlations with changes in relative abundance of specific taxa. Connected subnetworks imply not just single metabolites, but partial or complete biochemical pathways. These connected subnetworks were generated by identifying all edges in the network that connect pairs of metabolite nodes where both nodes' PRMT-scores significantly correlate with the relative abundance of a specific bacterial phylum (Figure 3). The largest connected subnetwork for interactions that correlated with specific phyla was comprised of 28 metabolites and 70 enzyme reactions, and was comprised of metabolic interactions associated with the metabolism of phosphonoacetate. PRMT-scores for metabolites in this largest subnetwork strongly correlated with relative abundances of *Planctomycetes *and *Gammaproteobacteria*. In the set of connected subnetworks were 5 small subnetworks of 5 or 6 nodes, each with PRMT-scores that exclusively correlated to the relative abundance of *Lentisphaerae*. Predicted metabolites in these subnetworks were associated with flavanoids and anthocyanins. Flavonoids have been observed to have antimicrobial effects on marine microorganisms [28] and flavonoid biosynthesis can be hypothesized to either have defense-related functions in *Lentispharae *or else other marine microorganisms possess the capacity to synthesize flavonoids in response to increased relative abundances of *Lentispharae*. Biosynthesis of terpanoids from the precursor Farnesyl pyrophosphate correlated strongly, not only with the relative abundances of *Crenarchaeota *and *Chlamydiae*, but also with the relative change in concentration of TOC. Terpenoids are a functionally diverse set of molecules whose synthesis by marine bacteria has been previously reported [29,30].

### Specific Examples of PRMT Analysis: primary production, phosphonate metabolism, and chitin catabolism

**Figure 4:**
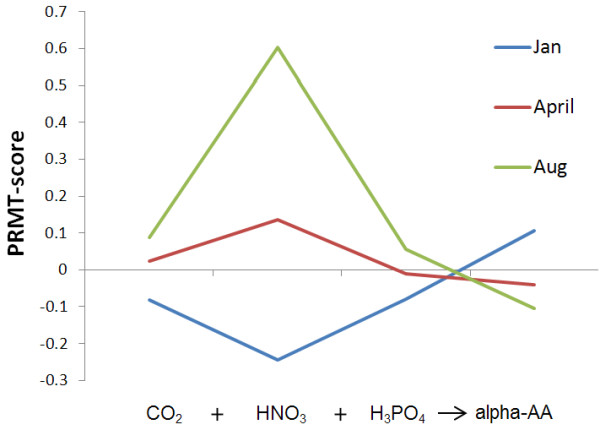
**PRMT-scores for photosynthesis pathway**. PRMT-scores for metabolites along the photosynthesis pathway are grouped by time point of metagenomic sample. In this figure, the marine biomass in equation for average photosynthesis is represented by PRMT-score for alpha-amino acids. A positive PRMT-scores indicate a predicted increase in relative catabolism, and negative PRMT-scores indicate a predicted increase in synthesis of a metabolite.

*Primary Production*: The chemical equation for average photosynthesis [31] provides a framework for analysis of PRMT-scores in the context of primary production:

We used PRMT-scores for the metabolite 'alpha-amino acids' to represent biomass in the above chemical equation. 'Alpha-amino acids' is a useful proxy for biomass as the single largest fraction of the dry-weight of prokaryote cells is protein [32]. The PRMT-scores for relevant metabolites in this equation indicate that, as expected, when CO_2_, SRP, and NO_3 _are consumed, more biomass (alpha-amino acids) is synthesized (Figure 4).

The PRMT scores for CO_2 _were most positive in August (indicating consumption) and most negative in January (indicating synthesis) (Figure 4). Also, PRMT scores indicated increased production of alpha amino acids (biomass) in summer, and active consumption in the winter **(**Figure 4). This matches expectations, as both bacterial and eukaryotic cellular biomasses are higher in summer [7]. When compared to investigating the abundance of sequences with homology to photosynthetic genes, which predict that the photosynthetic apparatus is more abundant in winter [4], PRMT scores reveal another level of potentially more useful information about the productivity of the ecosystem. While generated from the same data, consideration of sequence abundances and PRMT scores provide two unique perspectives. The largest PRMT-score shift in primary production was observed for nitrate. PRMT-scores for nitrite correlated strongly with the observed relative abundance of chlorophyll A. PRMT-scores for orthophosphate and alpha-amino acid all had strong correlations with PRMT-scores for CO_2_. As previously noted (Table 1), relative abundance of biological measurements of chlorophyll A had a strong correlation with PRMT-scores for chlorophyll A, linking the molecular components for photosynthesis with the PRMT-predicted capacity to synthesize the mechanism of photosynthesis.

One key limiting metabolite for primary productivity-derived biomass is iron [33,34]. Increased concentrations of Fe^2+ ^correlates with increased primary productivity in ocean microbial populations [35-37]. The PRMT-scores for Fe^2+ ^demonstrated a strong positive correlation with PRMT-scores for alpha amino-acids, the EMM proxy for biomass, as well as the relative abundance of measured TON. PRMT analysis identified a sound positive correlation between Fe^2+ ^and production of bacterial biomass.

*Phosphonoacetate metabolism*: Soluble Reactive Phosphorus (SRP) is considered to be a limited nutrient in marine ecosystems, although this is probably not the case in the Western English Channel [38]. However, it was interesting that PRMT scores for inorganic phosphate (orthophosphate) were strongly negatively correlated (PCC -0.93) with changes in the relative concentration SRP (Table 1). One reason for this discrepancy is that SRP is comprised of both inorganic and organic phosphate compounds. One group of organic phosphate compounds, the phosphonates, were considered recalcitrant to biological life for many years, but recent evidence from the Western English Channel suggests that they are used by a wide variety of bacteria and constitute a large fraction of the available phosphorus pool [38]. Currently, it is not technically feasible to measure specific phosphonate concentrations in marine systems, and this makes computational approaches like PRMT potentially valuable for investigating their relative turnover. Strikingly, 92% of the enzyme activities in the phosphonate and phosphinate metabolism KEGG pathway (map00440) were represented in the predicted EMM, indicating that the metabolic capacity to utilize phosphonates was present in the metagenomes.

The PRMT-scores for phosphonate compound, 3-Phosphonopyruvate, had strong correlations with PRMT-scores for CO_2_, and with biological measurements of chlorophyll A, suggesting a potential relationship between phosphonopyruvate metabolism and primary production. This is not totally unexpected, as phosphonopyruvate is a key intermediate step in the biosynthesis of all known natural phosphonates [39]. Another phosphonate of potential interest is phosphonoacetate, as the presence of phosphonoacetate as a natural product has been suspected but not confirmed [40]. Measured phosphonoacetate was strongly correlated with relative abundances of *Planctomycetes*, *Gamaproteobacteria*, and *Betaproteobacteria*. This insight into metabolic pathways associated with a metabolite difficult to measure in the environment led to a study to screen for a phosphonoacetate-oxidising activity in marine microorganisms; such an activity has recently been identified in cell-extracts of *Roseovarius nubinhibens*, and is now being characterized [unpublished data].

*Chitin catabolism: *The highly insoluble, nitrogen-containing compound chitin is the most abundant polymer in the ocean [41-43]. Yet, it is so rapidly degraded to fructose-6-phosphate, acetate, and ammonia that traces of chitin are only found in marine sediments [44]. Marine bacteria were primarily responsible for this rapid turnover, and chitin catabolism in marine microorganisms has been well studied [45-48]. Therefore, PRMT analysis was used to test the hypothesis that chitin degradation is a likely source of ammonia in the Western English Channel. Chitin is degraded via the following pathway (KEGG amino sugar and nucleotide sugar metabolism pathway (map00520)):

These reactions were all represented in the Western English Channel metagenomes. In the KEGG metabolic pathways, Fructose-6P metabolism leads to KEGG pathways for Glycolysis/gluconeogenesis (map00010) and Fructose and mannose metabolism (map00051), where both acetate and ammonia are generated.

**Figure 5:**
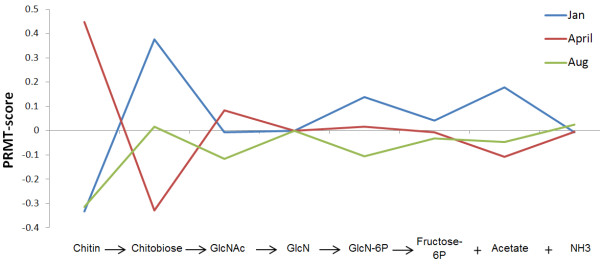
**PRMT-scores for chitin catabolism subnetwork**. PRMT-scores for metabolites along the chitin catabolism pathway are grouped by time point of metagenomic sample. Positive PRMT-scores indicate a predicted increase in relative catabolism, and negative PRMT-scores indicate a predicted increase in synthesis of a metabolite.

When considering PRMT-scores for the metabolites on the pathway for chitin degradation (Figure 5), a pattern for chitin catabolism was evident. Relative turnover of chitin was highest in April, concurrent with the spring bloom of chitin-synthesizing diatoms in the Western English Channel [49]. Increased turnover of chitin in spring leads to a predicted relative increase in the synthesis of chitobiose and increased consumption of GlcNac, ultimately yielding increased relative synthesis of chitin's breakdown products, Fructose-6P, acetate, and ammonia. Relative abundance of measured concentrations of ammonia had a strong negative correlation with PRMT-scores for GlcNAc. This indicates that when the environmental capacity for the consumption of GlcNAc was increased, the relative synthesis of ammonia was also increased. This was consistent with the reactants and products in the chemical equation for Figure 5. The PRMT-scores for GlcNAc had a strong positive correlation with the relative abundance of the Actinobacteria, members of which are involved in decomposition of organic materials such as chitin [50]. The PRMT-scores predicted an increase in the turnover of chitin, concurrent with spring diatom bloom and with increase in relative abundance of bacterial Phyla predicted to possess the capacity for chitin degradation. These PRMT scores and taxonomic correlations support the hypothesis that measured environmental concentrations of ammonia can be linked to bacteria catabolism of available large environmental pools of chitin.

## Conclusions

We have presented a novel computational approach, Predictive Relative Metabolic Turnover (PRMT), for making predictions of the relative change in the production or consumption of a metabolite by a microbial community. The set of all PRMT-scores for all the metabolites predicted to be present in an environmental metabolome provides a system-scale representation of the environmental metabolome derived from metagenomic sequence analysis that changes in response to fluctuations in the structure of its bacterial community. The approach was supported by comparing predicted relative turnover of metabolites to relative abundance of the biological measurements for those same metabolites. PRMT also provided the expected relationships between CO_2_, Iron, orthophosphate, nitrate, and chlorophyll with marine primary production. Specific, testable, biological hypotheses regarding the utilization of organic phosphorus and chitin were made, some of which are currently being actively investigated. The predictions made by PRMT are consistent with the hypothesis that bacterial population diversity is linked to the metabolic capacity of the community. While we have restricted our analysis to the metagenome, PRMT calculations are equally applicable to metatranscriptomic data. Ongoing environmental monitoring projects such as the Global Ocean Survey, TARA Oceans, Hawaiian Ocean Time Series, Bermudan Ocean Time Series, the Long Term Ecological Research sites, NEON, and the extended application of the Earth Microbiome Project http://www.earthmicrobiome.org are generating vast amounts of metagenomic and experimental metadata that can readily be investigated by further PRMT analyses.

## Abbreviations

PRMT: Predicted Relative Metabolomic Turnover; EMM: Environmental Metabolomic Matrix; EAC: Enzyme Activity Count; nEAC: Normalized Enzyme Activity Count; TOC: Total Organic Carbon; TON: Total Organic Nitrogen; SRP: Soluble Reactive/Phosphate; PCC: Pearson Correlation Coefficient.

## Competing interests

The authors declare that they have no competing interests.

## Authors' contributions

Conceived and designed the experiments: PEL, JAG. Performed the experiments: JAG, DF, PEL. Analyzed the data: PEL, JM, JQ, JAG. Contributed reagents/materials/analysis tools: FRC, FM, JAG. Wrote the paper: PEL, FRC, JAG, KPK, CSH, JM, JQ, DF. All authors read and approved the final manuscript

## Supplementary Material

Additional file 1 **Table S1**. This file contains a list of all enzymatic reactions in the predicted environmental metabolome for the Western L4 Station. Each line in the tab-separated file is a metabolic reaction in the format: Reactant, unique enzymatic function, product. Click here for file

Additional file 2 **Figure S1**. The network and calculated PRMT-scores. Click here for file

Additional file 3 **Table S2**. This file contains all calculated PRMT-scores, all PCC-scores between PRMT and relative abundances of Taxa, and between PRMT and relative abundances of measured environmental metabolites for the Western English Channel L4 Station environmental metabolome. Click here for file
